# Effects of Supplementation and Training on Ameliorating Lipid Profiles and Protection against Coronary Artery Disease; an Experimental Study

**Published:** 2019-02-12

**Authors:** Reza Vafaee, Hamid Soori, Mehdi Hedayati, Hamid Reza Hatamabadi

**Affiliations:** 1 Safety Promotion and Injury Prevention Research Center, Student Research Committee, Shahid Beheshti University of Medical Sciences, Tehran, Iran.; 2 Proteomics Research Center, Shahid Beheshti University of Medical Sciences, Tehran, Iran.; 3 Safety Promotion and Injury Prevention Research Center, School of Public Health, Shahid Beheshti University of Medical Sciences, Tehran, Iran.; 4 Cellular and Molecular Endocrine Research Center, Research Institute for Endocrine Sciences, Shahid Beheshti University of Medical Sciences, Tehran, Iran.; 5 Safety Promotion and Injury Prevention Research Center, Department of Emergency Medicine, Imam Hossein Hospital, Shahid Beheshti University of Medical Sciences, Tehran, Iran.

**Keywords:** Resveratrol supplementation, lipid profiles, Wistar rat, endurance and acute exercise trainings

## Abstract

**Introduction:**

The use of antioxidants may reduce the harmful effects of radicals during exercise and extreme sports. The Current study aimed to investigate the effect of this supplement on the lipid profiles in exercise-induced muscle injury.

**Methods:**

In this experimental study, 64 Wistar rats were randomly divided into four groups of control, exercise, exercise + Resveratrol (REV) and REV. After a week of adaptation, endurance and acute exercises were conducted in a motor driven treadmill, followed by using a training protocol in which running speed was gradually elevated until 19 weeks of age. Finally, the levels of cholesterol (CHO), triglycerides (TG), low-density lipoproteins (LDL), high-density lipoproteins (HDL), and very low-density lipoproteins (VLDL) were compared between the groups.

**Results:**

There was no statistically significant difference in CHO plasma level between the studied groups after acute and endurance exercises. There was a significant increase in the level of TG in the exercise group (p = 0.001) and the exercise + REV (p = 0.004) group after acute and endurance exercises. After the implementation of the endurance and acute exercises none of the studied groups had statistically significant changes in HDL plasma level. There was a significant decrease in LDL plasma levels in the exercise (p = 0.007) and the exercise + REV (p = 0.01) groups. After performing endurance protocol, VLDL plasma levels increased significantly in the exercise (p = 0.001) and the exercise+ REV (p = 0.005) groups in comparison with control group.

**Conclusions:**

Based on the findings, there was no difference in the level of CHO and HDL between the training groups, REV and control groups. However, both endurance exercise and acute exercise trainings resulted in an increase in TG and VLDL levels and decrease in LDL level, compared with the control group.

## Introduction

Cardiovascular disease will cause more than 75% of deaths worldwide by 2020, if this trend continues, about 24.4 million people will die before 2030 (1, 2). Physical inactivity emphasizes adverse outcomes such as atherosclerosis, diabetes, obesity, and metabolic syndrome. On the other hand, sports activities can be capable of reducing and preventing such diseases (3, 4).

One of the most sensitive targets for peroxidants can be non-saturated fatty acids in biological membranes that interfere with the pathogenesis of many diseases. Furthermore, cellular toxicity metabolites from lipid peroxides play a significant role in the oxidation of proteins present in low-density lipoprotein (*LDL*). This trend is also important in the pathogenesis of atherosclerosis (5).

Physical activity can lead to a 1.8-fold increase in lipid peroxidation after 60 minutes of intense cycling exercise. On the other hand, the implementation of intense and prolonged exercise can also lead to functional impairment of immunity, inflammation, oxidative stress and muscle damage (6, 7). Exercise-induced oxidative stress may be neutralized with antioxidants. Antioxidants, even at very low concentrations, along with an oxidizing agent, prevent oxidation of this oxidizing agent and are capable of delaying it (8, 9).

The use of antioxidants improves the antioxidant status of the body and may reduce the harmful effects of radicals during exercise and extreme sports (9, 10).

Resveratrol (REV) has been introduced for the first time in the 1940s in the Cassia Quniquangulata. It has many therapeutic aspects and has been previously used in Iran since around 6,000 years BC (11). The Chinese and Japanese used a plant called Polygonum Cuspidatum in the year 100 BC to treat many diseases. In the 1960s, Rev was isolated from this plant (12). REV is a specific polyphenol compound and its antioxidant nature is high due to the presence of two phenolic rings on both sides of the double bond; indeed, this stilbenoid is one of the most potent antioxidants found in nature (13, 14).

A number of reports suggested that co-administration of REV with statins was capable of increasing the cardio-protective effect of patients with cardiac arrhythmias. Chen et al. revealed the inhibitory effect of REV on cardiac hypertrophy in 2008 (15). 

 The supplementation of REV in prevention and treatment of cardiovascular diseases was initially investigated by controlling LDL, which is the main cause of atherosclerosis progression (16). REV protects the heart against myocardial damage following ischemia, and it has been shown that the supplementation of REV has improved cardiac function in hypertension induced in laboratory rats (17). Considering the positive effect of REV on inhibiting oxidative stress and inflammatory response from free radicals, the present study was aimed to investigate the effect of this supplement on the lipid profiles in exercise-induced muscle injury.

## Methods

***Study design and setting***

This experimental study was performed in Shahid Beheshti University of Medical Sciences from February 2017 to March 2018. Male Wistar rats were randomly divided into four groups: control, exercise, REV, and exercise + REV and their lipid profiles were compared after interventions. All animals were enrolled in the study according to approved guidelines for care and use of laboratory animals. They were given a standard commercial diet that was purchased from Pars Animal Feed Company, Iran. The protocol of the study was approved by the local ethical committee (IR.SBMU.MSP.1396.372).

***Participants***

Sixty-four male Wistar rats (165–175 g; 6-weeks-old) were obtained from Razi Research Institute of Karaj, Iran, and housed into standard conditions in groups of 4 animals per cage (18”x 10”x 8”) under a 12 h light/dark cycle, at 22 ± 3°C, with a 45% relative humidity. Overall, a total of 16 male Wistar rats per group were included in the present study.

***Intervention***

Male Wistar rats in trained group were subjected to the familiarization step for one week exercising on a rodent treadmill for 10 minutes, three times a week, in which the running speed was set to 5 to 10 m/min. Additionally, the control groups had access to the treadmill running three days during the week for 10 minutes, where exercise session was planned to be constant at a slow speed with an electrical stimulator. REV supplementation (10 mg/kg of REV in ethanol 2%/100 mL H2O) was began during exercise. On the other hand, those in the control group were also administered with water-containing ethanol 2% /100mL H2O as the vehicle.

***Exercise protocol***

After a week of adaptation, the rats began performing the protocol at 8 weeks of age. The training included endurance and acute exercises, which were conducted in a motor-driven treadmill at a speed of 10 m/minute, 20 min/day for 5 days/week, followed by applying a training protocol in which running speed was gradually elevated to 30 m/min for 60 min/day until 19 weeks of age. It is noteworthy that exercise intensity was adjusted to 65% maximal oxygen uptake (VO_2_max) as described previously (18).

**Table 1:** Changes in plasma levels of cholesterol (CHO), triglyceride (TG), high density lipoprotein (HDL), low density lipoprotein (LDL) and very low density lipoprotein (VLDL) after endurance exercise

Group	CHO	P	TG	P	HDL	P	LDL	P	VLDL	P
C vs. T	51.61(38.35) 56.37(9.5)	0.96	65(22.07) 119.12(23.48)	<0.001	33.5(1.64) 36.5(2.44)	0.42	33.66(6.88) 19.62(6.67)	0.007	13(4.41) 23.82(4.69)	<0.001
C vs. S	51.61(38.35) 57.71(6.62)	0.94	65(22.07) 76.85(15.42)	0.66	33.5(1.64) 37.57(5.06)	0.2	33.66(6.88) 29.57(7.2)	0.74	13(4.41) 15.37(3.08)	0.66
C vs. T+S	51.61(38.35) 55.85(10.62)	0.97	65(22.07) 104.57(10.34)	0.004	33.5(1.64) 38.28(4.07)	0.1	33.66(6.88) 20.28(8.11)	0.01	13(4.41) 20.9(2.08)	0.005
T vs. S	56.37(9.5) 57.71(6.62)	0.99	119.12(23.48) 76.85(15.42)	0.001	36.5(2.44) 37.57(5.06)	0.93	19.62(6.67) 29.57(7.2)	0.06	23.82(4.69) 15.37(3.08)	0.001
T vs. T+S	56.37(9.5) 55.85(10.62)	1.00	119.12(23.48) 104.57(10.34)	0.45	36.5(2.44) 38.28(4.07)	0.77	19.62(6.67) 20.28(8.11)	0.99	23.82(4.69) 20.9(2.08)	0.44
S vs. T+S	57.71(6.62) 55.85(10.62)	0.99	76.85(15.42) 104.57(10.34)	0.04	37.57(5.06) 38.28(4.07)	0.98	29.57(7.2) 20.28(8.11)	0.1	15.37(3.08) 20.9(2.08)	0.04

C = control group, T = train or exercise group, S = supplement or resveratrol, T+S= exercise + resveratrol.

**Table 2:** Changes in plasma levels of cholesterol (CHO), triglyceride (TG), high density lipoprotein (HDL), low density lipoprotein (LDL) and very low density lipoprotein (VLDL) after acute exercise

Group	CHO	P	TG	P	HDL	P	LDL	P	VLDL	P
C vs. T	40.71(8.51) 52(12.4)	0.32	67.57(14.5) 148.25(7.41)	<0.001	35.14(3.71) 37.5(3.87)	0.85	34.71(10.78) 15.5(7.54)	0.04	13.51(2.9) 29.65(1.48)	<0.001
C vs. S	40.71(8.51) 54.25(12.2)	0.08	67.57(14.5) 76.37(18.85)	0.68	35.14(3.71) 38.5(5.04)	0.53	34.71(10.78) 28.25(14.12)	0.67	13.51(2.9) 15.25(3.72)	0.68
C vs. T+S	40.71(8.51) 48.16(7.8)	0.57	67.57(14.5) 102.83(14.64)	0.002	35.14(3.71) 38(5.83)	0.7	34.71(10.78) 23.5(7.25)	0.28	13.51(2.9) 20.56(2.92)	0.002
T vs. S	52(12.4) 54.25(12.2)	0.98	148.25(7.41) 76.37(18.85)	<0.001	37.5(3.87) 38.5(5.04)	0.98	15.5(7.54) 28.25(14.12)	0.25	29.65(1.48) 15.25(3.72)	<0.001
T vs. T+S	52(12.4) 48.16(7.8)	0.93	148.25(7.41) 102.83(14.64)	0.001	37.5(3.87) 38(5.83)	0.99	15.5(7.54) 23.5(7.25)	0.67	29.65(1.48) 20.56(2.92)	0.001
S vs. T+S	54.25(12.2) 48.16(7.8)	0.7	76.37(18.85) 102.83(14.64)	0.02	38.5(5.04) 38(5.83)	0.99	28.25(14.12) 23.5(7.25)	0.85	15.25(3.72) 20.56(2.92)	0.02

C = control group, T = train or exercise group, S = supplement or resveratrol, T+S= exercise + resveratrol.

**Figure 1:**
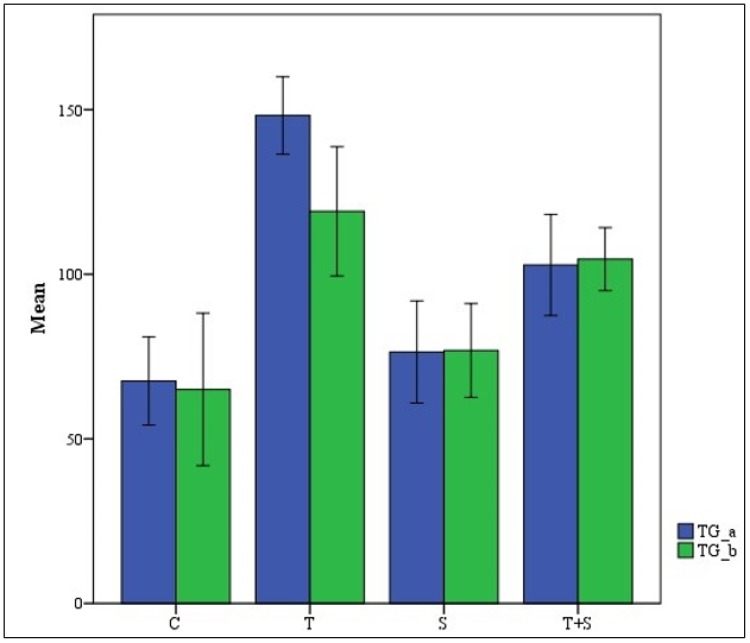
Comparison of plasma triglycerides changes in acute exercise (a) and endurance exercise (b) groups. C = control group, T = train or exercise group, S = supplement or resveratrol, T+S= exercise + resveratrol. Error bar = 95% confidence interval

The animals were sacrificed intra-peritoneally with 30-50 mg/kg ketamine and 3-5 mg /kg xylosin three days after the last exercise procedure for avoiding acute metabolic effects induced by the final run. 

Between 7 and 10 ml of blood samples were obtained at sacrifice by cardiac puncture in a heparin-treated 10-ml syringe. After that, all samples were centrifuged for 15 min at 3000 x g at 4°C, and serum samples were immediately stored at −20°C until use. Lipid profiles including cholesterol (CHO), Triglycerides (TG), low-density lipoproteins (LDL), high-density lipoproteins (HDL), and very low-density lipoproteins (VLDL) were biochemically measured using commercially available *kits* according to the *manufacturer's protocols*. 

***Statistical Analysis***

The data was collected by the first author. All Data were processed and analyzed using IBM SPSS Statistics ver. 21.0. For statistical evaluation of the changes in all groups, we applied one-way analysis of variance (ANOVA). Statistical significance was set at P<0.05. Additionally, Tukey's test was applied for post-hoc comparisons.

## Results

Tables 1 and 2 compare the changes of lipid profile between different groups after acute and endurance exercises.

***CHO plasma levels***

There was no statistically significant difference in CHO plasma level between the studied groups after acute and endurance exercises (Tables 1 and 2).

***TG plasma levels***

Following the implementation of the endurance exercise protocols, there was a significant increase in the level of TG in the exercise group (p = 0.001) and the exercise+ REV (p = 0.004) group compared to the control group, but no significant difference was found in terms of plasma TG levels between the exercise and exercise + REV groups (p = 0.450; Table 1).

On the other hand, a statistically significant increase was observed in plasma TG levels in the exercise (p < 0.001) and exercise + REV (p = 0.002) groups compared with the control group after performing acute protocol. However, the plasma TG levels in the REV group demonstrated a significant decrease compared to the exercise group (p < 0.001; Table 2). There was a significant difference between the TG plasma level of exercise group after acute and endurance exercises (p = 0.03, figure 1).

***HDL plasma levels***

As shown in tables 1 and 2; after the implementation of the endurance and acute exercise none of the studied groups had statistically significant changes in HDL plasma level. Also, no statistically significant difference was revealed in the HDL plasma level between the groups undergoing endurance and acute exercises. 

***LDL plasma level***

There was a significant decrease in LDL plasma levels in exercise (p = 0.007) and exercise + REV (p = 0.01) groups compared to control group. After performance of the acute exercise, plasma LDL level showed a significant decrease in the exercise group compared with the control group (p = 0.049). There was no statistically significant difference in the LDL plasma levels between endurance and acute exercise groups.

***VLDL plasma levels***

After performing endurance protocol, VLDL plasma levels increased significantly in the exercise (p = 0.001) and the exercise+ REV (p = 0.005) groups in comparison with control group. After implementation of the acute protocol, there was a significant increase in plasma VLDL level in the exercise group (P < 0.0001) and the exercise + REV group (p = 0.002) when compared with the control group. However, VLDL plasma levels in the exercise +REV exhibited a statistically significant difference compared to the exercise group (p = 0.001). Furthermore, the VLDL plasma level did not reveal a significant difference between endurance and the acute exercises. 

## Discussion:

Based on the findings, there was no difference between the training groups, REV and control groups regarding the level of CHO and HDL. However, both endurance exercise and acute exercise trainings resulted in an increase in TG and VLDL levels and decrease in LDL level in comparison with the control group. 

A growing body of evidence has shown that regular exercise with moderate intensity prevents cardiovascular disease due to increased HDL level in the blood. Regular exercise boosts the antioxidant system and prevents cardiovascular disease in the long run. 

Santin et al. reported that levels of CHO and LDL decreased in rats with mild exercise, but no change was found in levels of HDL and TG (19). REV exerts many effects, such as antioxidant activity, regulation of lipid and lipoprotein metabolism, inhibition of platelet aggregation, and *vasodilation* (20, 21).

REV, as the activator of the enzyme AMPK, plays a role in regulating lipid metabolism and prevents the accumulation of lipid in the cells, where it has been revealed that a synthesized derivative of REV was capable of enhancing AMPK phosphorylation, and reducing hepatic TG accumulation, showing a significant therapeutic effect on fatty liver disease (22).

In the study by Kitada et al., oral administration of Risoratrol to diabetic mouse models improved their lipid profiles (23). In other words, the aforementioned study indicated that REV could be an effective supplement for improving renal injury and was capable of increasing mitochondrial biogenesis with Mn-SOD dysfunction in diabetic mouse models by improving oxidative stress, as well as normalization of the Mn-SOD function and glucose-lipid metabolism, suggesting an anti-oxidative activity for REV by affecting AMPK/SIRT1-independent pathway(23).

Castro et al. found that REV could play a significant role in preventing LDL deposition in the aortic artery endothelium and accelerating its recovery, showing a preventive effect in development of atherosclerotic lesions (24). The findings of the present study are consistent with the findings of Kitada et al. (23) and Zhu et al. (25), in which this combination was involved in improving lipid profiles. Our results also exhibited a significant decrease in TG and VLDL levels in the exercise group + REV compared to the training group. However, some believe that improvement of coronary heart diseases by REV is not due to its antioxidant property against lipids and changing lipoprotein profile (26). Zhu et al. also demonstrated both antioxidant and anti-hyper-lipidemic effects for REV. They showed that administration of REV in hyperlipidemia rats has a significant effect on lipid profiles and plays a role in decreasing lipid profile (TG and CHO levels) by decreasing hepatic thiobarbituric acid reactive substances via inhibition of the oxidation of LDL (25).

On the other hand, Tomé-Carneiro et al. reported that a grape extract supplement containing REV led to a decrease in the level of oxidized LDL and apolipoprotein-B in patients at high risk of a cardiovascular disease. This supplement was capable of decreasing atherogenic markers and was involved in cardioprotection (21). However, in the current study, the supplementation of REV did not change the level of LDL. This finding is incompatible with Tomé-Carneiro et al. (21). 

Overall, since REV reduces the level of TG, it seems that REV may show a protective effect against lipid peroxidation in athletes by improving the lipid profile and reducing peroxidation of lipids. However, these effects can be affected by several other factors such as type and duration of exercise, the type of model examined, the amount and timing of resveratrol supplementation and other factors. Therefore, further studies are required to clarify the effect of this supplement on lipid profiles during endurance and acute exercise.

## Conclusion:

Based on the findings, there was no difference in the level of CHO and HDL between the training groups, REV and control groups. However, both endurance exercise and acute exercise trainings resulted in an increase in TG and VLDL levels and decrease in LDL level, compared with the control group. 
